# Upregulation of musashi1 increases malignancy of hepatocellular carcinoma via the Wnt/β-catenin signaling pathway and predicts a poor prognosis

**DOI:** 10.1186/s12876-019-1150-6

**Published:** 2019-12-30

**Authors:** Qiuhua Liu, Cuijie Zhou, Bo Zhang

**Affiliations:** Department of General Surgery, The First People’s Hospital of Zhangjiagang, The Affiliated Zhangjiagang Hospital of Soochow University, 68 West Jiyang Road, Zhangjiagang, Jiangsu 215600 People’s Republic of China

**Keywords:** musashi1, Biomarker, Hepatocellular carcinoma, Malignant transformation, Wnt/β-catenin signaling pathway, Target therapy

## Abstract

**Background:**

Hepatocellular carcinoma (HCC) is a common human malignant cancer due to a high metastatic capacity and the recurrence rate is also high. This study is aim to investigate the role of musashi1 as a potential biomarker for therapy of HCC.

**Methods:**

The mRNA and protein expression levels of musashi1 were detected in HCC samples and cell lines. The malignant properties of HCC cells, including proliferation, invasion and migration were measured by overexpressing or knocking down expression of musashi1. Additionally, the correlation between musashi1 and clinicopathological indexes and prognosis were analyzed. The expression of CD44 was measured and the correlation between CD44 and musashi1 was analyzed.

**Results:**

In vitro cytological experiments demonstrated that musashi1 was elevated in HCC samples and cell lines and this increased expression affected cancer cell viability, migration and invasive capacity by activating of the Wnt/β-catenin signaling pathway. Analysis of clinicopathological characteristics suggested that up-regulation of musashi1 was related to metastasis potential and a poor prognosis. Besides, there was a positive correlation between CD44 and musashi1 expression. Upregulation of musashi1 in malignant liver tumors may have contributed to the maintenance of stem-cell like characteristics of HCC cells.

**Conclusions:**

Upregulation of musashi1 could enhance malignant development of HCC cells and thus might be a novel marker for HCC therapy.

## Background

Primary liver cancer (PLC) which with a high morbidity and mortality rate, is the third leading cause of cancer-associated death all over the world [1, 2]. Hepatocellular carcinoma (HCC) accounts for 70–85% of cases of PLC based on histological classification [3]. Although several improvements have been made in the diagnosis and treatment of HCC in recent decades, the high recurrence and metastatic rate seriously reduces prognosis [4]. Examining the mechanisms underlying recurrence and metastasis may assist in limiting tumor malignant progression, improve the prognosis and survival of patients. But the reality is, the specific molecular mechanisms underlying HCC malignant development still unknown. Devepment of new valuble targets may provide potential targets for preventing HCC invasion and metastasis.

Musashi1 was initially discovered in Drosophila and is essential for the development of adult external sensory organs. Musashi1 contains an conserved RNA binding protein domain and is used as a molecular marker of neural stem cells [5, 6]. Several reports have demonstrated musashi1 is abnormally expressed in a variety of malignant tumors, including retinoblastoma, lung cancer, esophageal adenocarcinoma, glioma and gastrointestinal cancer [7–11], and in these malignant tumors, musashi1 is associated with tumor stem cells and affects the recurrence and metastasis of tumors. It is hypothesized that tumor stem cells are an important source of maintaining the growth of tumor tissues, as they self-renew and are pluripotent in their differentiation capacity [12, 13]. However, whether musashi1 maintains a stem cell phenotype and promotes malignant transformation of HCC cells remains unclear.

Based on our present study, the expression pattern of musashi1 in HCC samples and cell lines were profiled. Furthermore, the mechanism by which musashi1 affected biological phenotype of HCC cells was determined and the prognostic significance of musashi1 was evaluated.

## Methods

### Patients and tissue specimens

Clinical samples were collected between April 2010 and February 2012. Tissue chip contained 67 HCC patients (tumor tissues and matched adjacent non-tumor hepatic tissues) were made. The diagnosis of HCC was confirmed based on postoperative pathology for each patient. All patients recruited in this study had not undergone any treatment before operation. Clinical follow-up continued until March 2017. This study was approved by the Hospital’s Research Ethics Committee and written informed consent was signed by all patients prior to surgery. The clinical data of the patients with HCC are summed up in Table 1.

**Table 1:** Correlation of Musashi-1 expression in HCC tissues with its clinical characteristics

Varriable	N (67)	Musashi-1 expression		χ^2^ value	*P* value
		Low (27)	High (40)		
Gender				0.011	0.915
Male	55	22	33		
Female	12	5	7		
Age, years				0.298	0.585
≤ 50	30	11	19		
>50	37	16	21		
HBsAg				1.513	0.219
Negative	17	9	8		
Positive	50	18	32		
AFP, ng/mL				3.118	0.077
< 20	15	9	6		
≧20	52	18	34		
No. of tumors				0.436	0.509
Single	29	13	16		
Multiple	38	14	24		
Tumor size, cm				2.053	0.152
≤ 5	23	12	11		
>5	44	15	29		
Tumor encapsulation				3.522	0.061
Complete	28	15	13		
None	39	12	27		
Tumor differentiation				4.204	0.040
Poor	22	5	17		
Well	45	22	23		
Vascular invasion				4.376	0.036
No	27	15	12		
Yes	40	12	28		
Intrahepatic metastasis				5.070	0.024
No	31	17	14		
Yes	36	10	26		
Extrahepatic metastasis				5.713	0.017
No	49	24	25		
Yes	18	3	15		

### Cells and vectors

The normal liver cell line WRL68 and HCC cell lines Hep3B, Huh7, PLC/PRF/5 and SMMC-7721 were purchased from the Cell Bank of Type Culture Collection of the Chinese Academy of Sciences and were routine cultured in DMEM, supplemented with 10% FBS (both from Invitrogen; Thermo Fisher Scientific, Inc).

The DNA fragments which encoded musashi1 were amplified by PCR and cloned into the multiple cloning sites of the expression vector pDC315 to generate pDC315-MSI1. The empty pDC315 vector was conducted as negative control. The plasmids containing short hairpin (sh) RNAs targeting musashi1 or scrambled shRNA control were purchased from OriGene Technologies Inc. and transfected into cells by using Lipofectamine® 2000 (Invitrogen; Thermo Fisher Scientific. Inc.).

### Immunohistochemistry (IHC)

All tissues were obtained from resected specimens during operation. Tissue chip were used to examine Musashi1 expression levels. After deparaffinization, rehydrated and microwaved to retrieve antigens, every slide was incubated with rabbit monoclonal anti-musashi1 primary antibody at 4 °C overnight (cat. no. ab52865; Abcam; 1:100), followed by incubation with the corresponding secondary antibodies at 37 °C for 30 min. As the negative control, tissues were prepared in the same protocol without incubation with the primary antibody. After staining by using 3,3′-diaminobenzidine and Mayer’s hematoxylin, representative images were collected by a microscope. The evaluation were scored as the proportion of immune-positively stained area multiplied by intensity of staining. Immuno-positive staining was classified as follows: 0%, 0; 1–25%, 1; 26–50%, 2; 51–75%, 3; 76–100%, 4. The intensity of staining was classified as follows: 0, negative; 1, weak; 2, moderate; 3, intense. The tissues were evaluated by two experienced pathologists independently. The median IHC score (4.0) was selected as the cut-off value to define high / low expression.

### Real-time polymerase chain reaction (qRT-PCR)

Total RNA was conventionally extracted from tissue samples by using TRIzol® reagent (Invitrogen; Thermo Fisher Scientific, Inc.), and was reverse-transcribed into cDNA. The conditions for PCR were as follows: 95 °C for 4 min; followed by 40 cycles of 95 °C for 20 s, 56 °C for 35 s and 72 °C for 30 s. A total of three technical replicates were performed for each cDNA sample. β-actin was used as internal control. The data was calculated by using 2^-ΔΔCq^ normalisation method.

The used primers were: Musashi1 forward, 5′- GCTCGACTCCAAAACAATTGACC-3′ and reverse, 5′- GGCTGAGCTTTCTTACATTCCAC-3′; CD44 forward, 5′-ATCCAGGCAACTCCTAGTAGTACAACG-3′ and reverse, 5′-TGTCCCTGTTGTCGAATGGGAGTCTTC-3′; and β-actin forward, 5′-CCACCCATGGCAAATTTC-3′ and reverse, 5′-GCCCAGGATGCCCTTGA − 3′.

### Western blot analysis

Total proteins from tissue samples and HCC cell lines were conventionally extracted by using RIPA lysis buffer (Beyotime Institute of Biotechnology) supplemented with PMSF (1:100, Sigma-Aldrich; Merck KGaA). The extracted proteins from tissues and cell lysates were loaded on a 10% SDS-gel and resolved using SDS-PAGE. The resolved proteins were electrical transferred to PVDF membranes (PerkinElmer). After blocking in 5% non-fat dry milk for 1 h, incubated PVDF membrane in diluted primary antibodies at 4 °C overnight. Next day incubated PVDF membrane with the appropriate secondary antibody at room temperature for 2 h. The information of primary antibodies used were rabbit anti-human musashi1 (cat. no. ab52865; Abcam; 1:500), rabbit anti-human CD44 (cat. no. ab189524; Abcam 1:1000), anti-human cyclinD1 (cat. no. sc-450; Santa Cruz Biotechnology, Inc.; 1:1000), mouse anti-human DKK1 (cat. no. sc-374,574; Santa Cruz Biotechnology, Inc.; 1:1000), mouse anti-human APC (cat. no. sc-53,165; Santa Cruz Biotechnology, Inc.; 1:1000), rabbit anti-human phospho-β-catenin (cat. no. 4176, Cell Signaling Technology, Inc.; 1:1000) and rabbit anti-human β-catenin (cat. no. 8480, Cell Signaling Technology, Inc.; 1:1000). The secondary antibodies used were horseradish peroxidase-conjugated sheep anti-mouse immunoglobulin G (IgG) and sheep anti-rabbit IgG (Cell Signaling Technology, Inc.; 1:5000). Protein bands were visualized with ECL substrates (Pierce). GAPDH (Kangchen Bio-tech; 1:6000) was used as the loading control.

### Cell counting Kit-8 (CCK-8) assay

The effects of overexpressing and knocking down musashi1 on proliferation of HCC cells were measured using a CCK-8 testing kit (Beyotime Institute of Biotechnology). After transfection, SMMC-7721 and Huh7 cells (4 × 10^3^/100 μl per well) were resuspended and seeded in 96-well plates for 48 h. Subsequently, 10 μl of CCK-8 solution was added and cells were further incubated for 2 h (avoiding light). The result was measured at 450 nm by using a microplate spectrophotometer.

### Colony formation assay

Transfected HCC cells were collected and resuspended in 1.5 ml culture media supplemented with 0.45% agarose (Invitrogen; Thermo Fisher Scientific, Inc.). The cells were seeded in 6 well plates containing 1.5 ml culture media and agarose (0.75%). After conventionally cultured for 2 weeks, cell colonies in 6 well plates were stained with 0.005% crystal violet dyeing solution (Beyotime Institute of Biotechnology). The experiment was performed in triplicate with three wells per condition in each repeat.

### Transwell assays

SMMC-7721 and Huh7 were cultured and transfected with the indicated plasmid. The scrambled shRNA transfected cells were used as the control. Cells of control group were cultured simultaneously. Cell invasion assays were evaluated by using transwell chamber assay (Millipore). Matrigel (BD Biosciences) was laid at the bottom of the upper chamber for 45 min at 37 °C. A total of 4 × 10^5^/200 μl cells were resuspended and added to the upper chamber. Complete medium was supplemented to the upper chamber. After cultured for 48 h, removing residual cells in the upper chamber and cells passing through the chamber were stained with 0.1% crystal violet dyeing solution for 15 min. The number of cells were counted under the microscope. The experiment was repeated three times.

### Wound healing assay

Cells were resuspended and seeded in 6 well plates (4 × 10^5^ cells per well). When cell growth density reached to about 80%, created a straight scratch in the cell monolayer by using a sterile pipette tip. Removing the dead cells by PBS solution for three times. Representative photos were taken at 0, 24 and 48 h. Migration aera was calculated using ImageJ.

### Statistical analysis

Statistical analyses were performed using SPSS version 18.0. χ^2^ tests and Fisher’s exact tests were used compare the clinicopathological data. Kaplan-Meier analysis was used to estimate survival rates and a two-sided log-rank test was used to compare differences. In vitro data were analyzed using a one-way ANOVA. *P* < 0.05 was considered to indicate a statistically significant.

## Results

### Musashi1 is upregulated in HCC cells and tissues

**Fig. 1:**
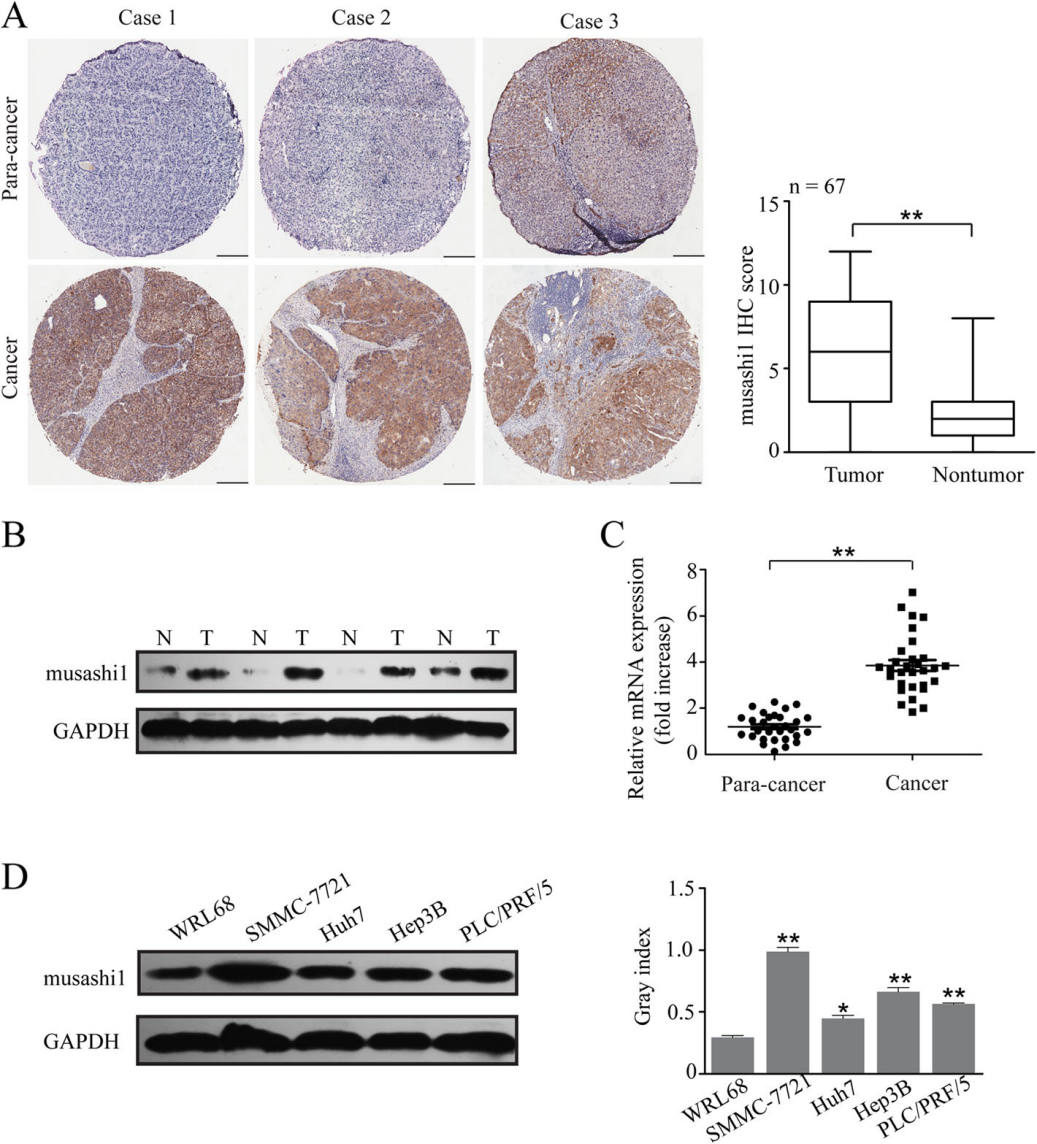
Expression patterns of musashi1 in HCC. **a** Expression of musashi1 in non-tumor and matching and HCC samples was measured by immunohistochemistry (IHC). Representative photos and the IHC scores are presented. Scale bar, 50 μm. **b** Protein expression of musashi1 in non-tumor and HCC tissues were evaluated by western blot assay and densitometry analysis was performed. **c** mRNA expression of musashi1 in non-tumor and HCC samples were examined by qRT-PCR. **d** Protein expression of musashi1 in the various HCC cells were examined and densitometry analysis was performed. ^*^P < 0.05, ^**^P < 0.001. HCC, hepatocellular carcinoma; IHC, immunohistochemistry

Previous studies reported that musashi1 expression was abnormally upregulated in cancers, including glioma, breast, colon and gastric cancer. Musashi1 was demonstrated to be a potential contributor of tumor development. However, the role of musashi1 in HCC still unclear. In our study the expression of musashi1 in 67 hepatoma cancer tissues and adjacent liver tissues were determined. The expression of musashi1 was significantly higher in 57 HCC cases (56/67, 83.6%; Fig. 1a). 30 cases of HCC tissues and adjacent normal liver tissues were selected for analysis of musashi1 mRNA and protein expression. Our results found that both mRNA and protein expression of musashi1 were significantly upregulated in HCC tissues (Fig. 1b and c; P<0.001), suggesting that musashi1 may be frequently upregulated in HCC tissues. In addition, the expression of musashi1 in HCC cell lines was evaluated by western blotting. As shown in Fig. 1d, compared with WRL-68 cells, the expression of musashi1 was upregulated in four HCC cell lines. According to this result, Huh7 and SMMC-7721 were selected for subsequent experiments. Together, these results suggested that musashi1 was overexpressed in HCC cells and tissue samples.

### Musashi1 promotes the proliferation and neoplastic capacity of HCC cells

**Fig. 2:**
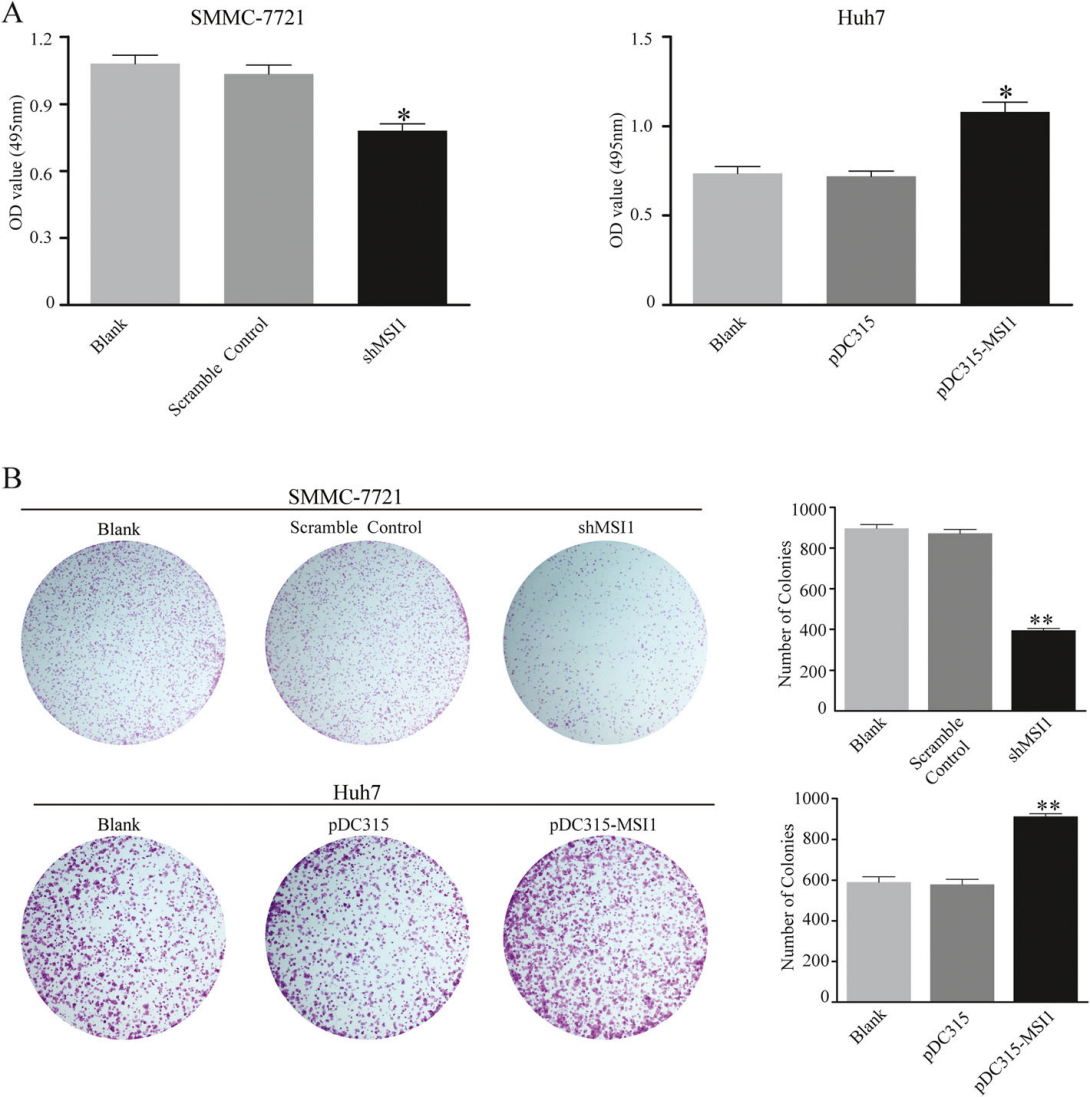
Musashi1 promotes the proliferation and neoplastic capacity of HCC cells. **a** Proliferative capacity of cells evaluated by using a CCK-8 testing kit. **b** Neoplastic capacity of transfected cells was detected using a colony formation assay. Representative images and the statistical analysis are presented. ^*^P < 0.05, ^**^P < 0.001

In order to examine the function of musashi1 on the proliferation of HCC cells, shRNA targeting musashi1 mRNA (shMSI1) or a musashi1 overexpression vector were transient transfected into SMMC-7721 and Huh7 cells to knockdown or overexpress musashi1, respectively. According to the different transfection efficiency of shRNAs targeting musashi1, shMSI1 was selected for subsequent experiment (Additional file 1: Figure.S1). SMMC-7721 and Huh7 cell proliferative activity was evaluated by using CCK-8 testing kit after transient transfection for 48 h. Our results showed that knockdown of musashi1 decreased cell viability of SMMC-7721 transfected with shMSI1 (Fig. 2a, *P*<0.05), and cell viability was significantly increased in Huh7 cells overexpressing musashi1 (P<0.05). The neoplastic capacity of HCC cells was examined using a colony-formation assay. The results demonstrated that fewer colonies were formed in the SMMC-7721-shMSI1 cells (P<0.001), and overexpression of musashi1 in Huh7 resulted in dramatically elevated colony formation capacity (P<0.001; Fig. 2b).

### Musashi1 increases the migratory and invasive capacity of HCC cells

**Fig. 3:**
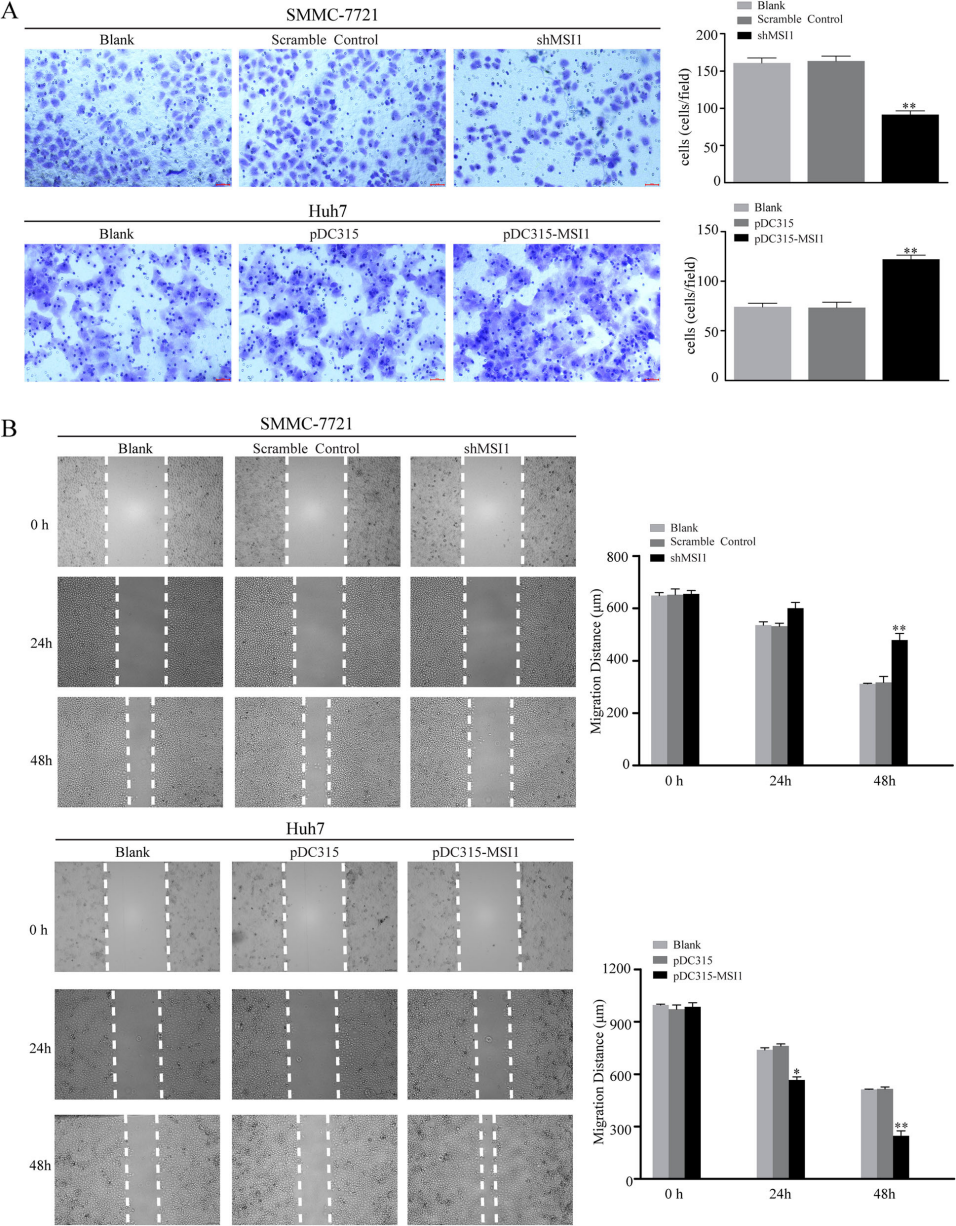
Musashi1 increases the migratory and invasive capacity of hepatocellular carcinoma cells. **a** Invasive capacity of transfected cells was detected using a Transwell invasion assay. **b** Migratory capacity of transfected cells was evaluated using a wound-healing assay. ^*^P < 0.05, ^**^P < 0.001

To determine the function of musashi1 on cell mobility of HCC cells, SMMC-7721 and Huh7 cells with musashi1 knocked down or overexpressed, respectively. The results of transwell assays showed that relative invasion capacity was significantly inhibited in the SMMC-7721-shMSI1 cells. Upregulation of musashi1 in Huh7 cells significantly promoted invasion capacity compared with the respective controls (Fig. 3a). Similarly, wound healing capacity was significantly inhibited in the SMMC-7721-shMSI1 cells; whereas, in the musashi1 overexpressing Huh7 cells, wound healing was significantly increased compared with the respective controls (Fig. 3b). Together, these findings suggest that musashi1 positively regulates cellular motility and invasion of HCC cells in vitro.

### Musashi1 regulates malignant transformation of HCC cells via the Wnt/β-catenin signaling pathway

**Fig. 4:**
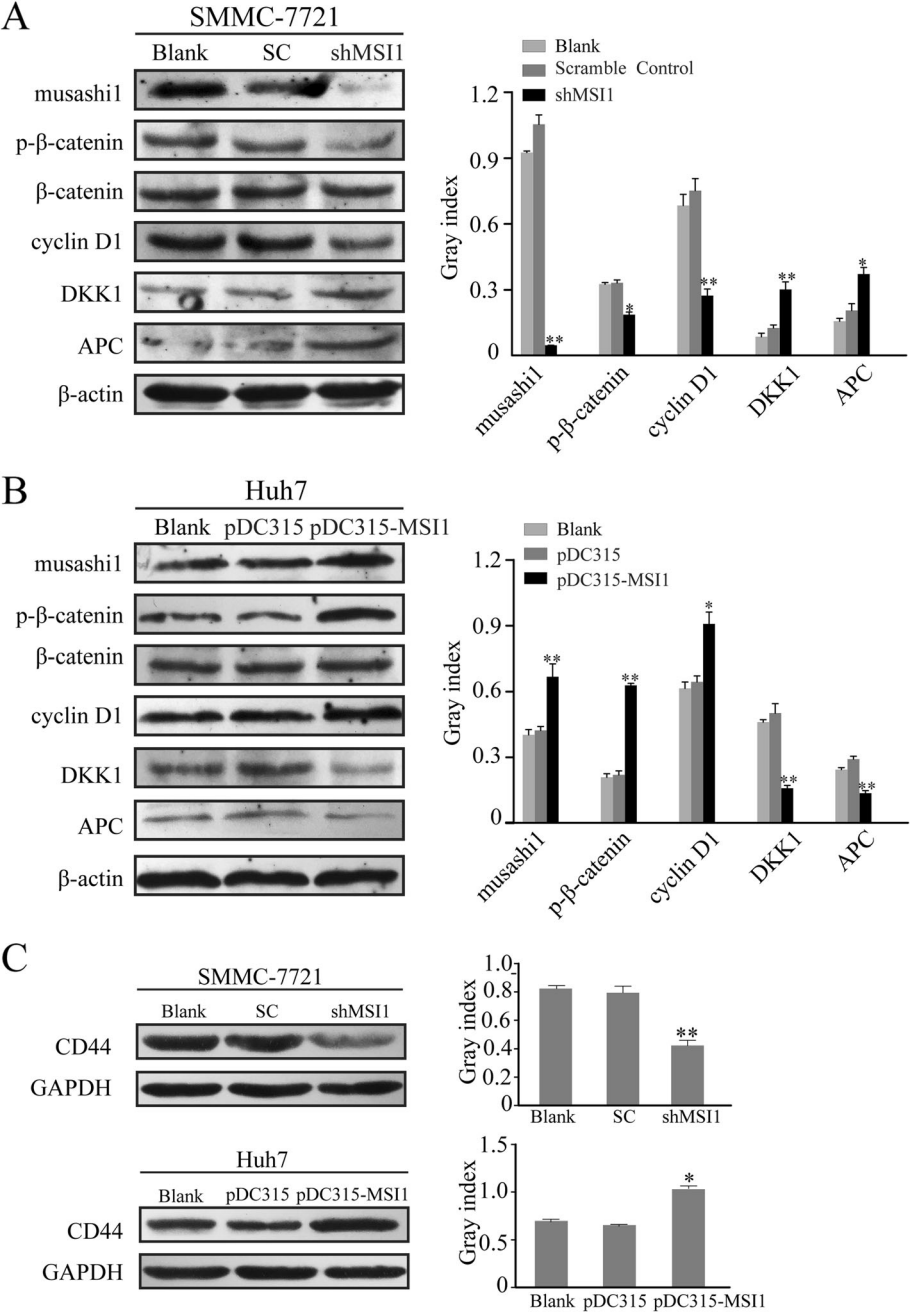
Musashi1 regulates malignant transformation via the Wnt/β-catenin signaling pathway. **a** and **b** Protein expression of musashi1, Wnt/β-catenin pathway associated proteins and cell cycle related proteins were measured by western blotting. Representative blots and the densitometry analysis are presented. **c** Protein expression levels of CD44 in HCC cells were evaluated by western blotting and densitometry analysis was performed. ^*^P < 0.05, ^**^P < 0.001

In order to investigate the specific molecular mechanisms underlying the functions of musashi1 in HCC, the relative expression levels of members of the Wnt/β-catenin pathway were measured by western blotting analysis. Our data demonstrated that downregulation of musashi1 in SMMC-7721 cells reduced the expression of phospho- (p-)β-catenin and cyclin D1 and elevated the protein expression of DKK1 and APC, which are key targets of the Wnt pathway. Conversely, the expression of p-β-catenin and cyclin D1 were upregulated, and this was accompanied by a reduction in DKK1 and APC expression in Huh7 cells overexpressing musashi1 (Fig. 4a and b). The results suggest that musashi1 overexpression may activate the Wnt/β-catenin pathway in HCC. In addition, the Wnt/β-catenin pathway may have served an important role in maintaining the stemness of HCC cells. As CD44 is a known cancer stem cell marker, the expression levels of CD44 in HCC cells were determined. A significant upregulation of CD44 expression was observed in Huh7 cells overexpressing musashi1 compared with the control cells. Additionally, knockdown of musashi1 decreased CD44 expression in SMMC-7721 cells compared with the control (Fig. 4c).

### Increased expression of musashi1 predicts a poor prognosis in patients with HCC

**Fig. 5:**
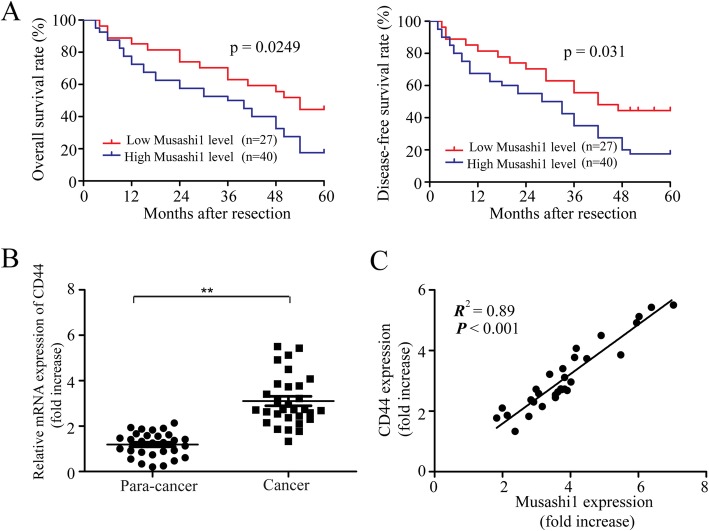
Increased expression of musashi1 predicts poor prognosis in HCC patients. **a** Correlation between musashi1 expression and prognosis of HCC patients by using Kaplan-Meier survival analysis. **b** mRNA expression levels of CD44 in non-tumor and HCC tissues. **c** Association between musashi1 and CD44 expression in HCC samples (*n* = 30). ^**^P < 0.001. HCC, hepatocellular carcinoma

The correlation analysis between musashi1 expression and the clinicopathological factors of HCC patients were determined. Statistical analysis suggested that high levels of musashi1 expression was closely related to tumor differentiation (*P* = 0.04), vascular invasion (*P* = 0.036), and intrahepatic (*P* = 0.024) and extrahepatic metastasis (*P* = 0.017) (Table 1). To examine the clinical outcomes of each HCC subtype identified above, Kaplan-Meier survival analysis was used. As shown in Fig. 5a, high levels of musashi1 expression was closely related to lower overall survival (log rank = 5.03, *P* = 0.0249) and recurrence-free survival (log rank = 4.454, *P* = 0.031) compared with patients with low levels of musashi1 expression in HCC patients. Therefore, abnormally overexpression of musashi1 was closely related to tumor metastasis and malignant transformation of HCC.

In addition, CD44, a known stem cell marker was measured by RT-qPCR in 30 pairs of liver tissues. Results suggested that the mRNA expression of CD44 were significantly upregulated in HCC tissues, compared with the corresponding normal liver tissue (Fig. 5b). Furthermore, a statistically significant positive association between CD44 and musashi1 expression was observed (Fig. 5c). These data revealed that abnormally upregulation of musashi1 in malignant liver tumors may contribute to maintenance of stem-cell like characteristics of HCC cells.

## Discussion

The musashi family of proteins are a conserved group of neural RNA-binding proteins. Musashi1 is selectively expressed in neural stem/progenitor cells and downregulated in differentiated cells [14, 15]. More and more reports have showed that musashi1 is abnormally overexpression in cancer stem cells [16–19] and musashi1 acts as a transcriptional regulator which changes the expression of its associated genes to maintain stem cell-like characteristics and self-renewal capacity. The expression levels of musashi1 are also a potential factor for evaluating the prognosis of different types of malignant tumors [20–22].

In this study, our results demonstrated that the expression (both mRNA and protein levels) of musashi1 were elevated in HCC, and upregulation of musashi1 was closely related to poor prognosis in patients with HCC. Besides, upregulation of musashi1 was associated with tumor differentiation, vascular invasion, intrahepatic metastasis and extrahepatic metastasis. Patients classified as having high levels of musashi1 expression had both reduced disease-free survival and a worse overall survival compared with the patients with lower expression levels. Our results suggested that musashi1 is associated with tumor metastasis, recurrence and malignant transformation. In addition, the expression of CD44, a marker of tumor stem cells, was overexpressed in HCC tissues in which expression of musashi1 was upregulated, and there was a dramatic correlation between expression of CD44 and musashi1. Therefore, it may be hypothesized that musashi1 may serve an important role in the maintenance and self-renewal capacity of liver cancer stem cells.

In vitro assays revealed that downregulation of musashi1 decreased proliferation, and the invasive and migratory capacity of HCC cells; whereas, overexpression of musashi1 increased the malignant behaviors of HCC cells. Therefore, it was hypothesized that musashi1 may promote malignant transformation of HCC cells. As a biomarker of tumor stem cells, musashi1 expression regulated cell cycle progression and apoptosis of endometrial cancer cells through the Notch1 signaling pathway [23]. A recent study showed that musashi1 serves an important regulatory role in chemotherapy and radiotherapy tolerance in the treatment of patients with pancreatic cancer [24]. In this study it was demonstrated that Wnt/β-catenin pathway was abnormally activated in HCC by overexpressing musashi1. To function as an RNA-binding protein, musashi1 acts as a transcriptional regulator which regulates the function of specific mRNAs. In the cytoplasm, musashi1 targets key genes in the Wnt/β-catenin pathway, affecting the transcription and post-transcriptional translation of specific genes, thereby disturbing its regulatory biological function. Abnormal activation of the Wnt/β-catenin pathway promoted the malignant capacity of cancer cells, including proliferation and invasion by inducing EMT process (epithelial-mesenchymal transition) [25]. Additionally, the Wnt/β-catenin signaling pathway participates in maintaining stem cell characteristic in normal cells and cancer cells [26]. CD44, a stem cell marker, was also elevated in cancer tissues with upregulated expression of Musashi1, and there was a positive association between them, suggesting that Musashi1 may serve a crucial role in the maintenance of the self-renewal capacity of HCC cells. These results suggested that musashi1 may maintain the self-renewal ability of cancer stem cells, which underlie malignant transformation. Additionally, musashi1 may promote progression of HCC by activating the Wnt/β-catenin pathway.

To sum up, musashi1 was abnormally expressed in tumors and it promotes carcinogenesis and chemo/radio-resistance. Therefore, musashi1 could be a novel therapeutic target for clinical treatment of HCC. Future efforts should focus on the specific molecular mechanisms underlying musashi1 upregulation, and development of synergistic combinations between direct or indirect inhibitors of musashi1 as an adjuvant treatment for patients undergoing chemo/radio-therapy to improve patient outcomes.

## Conclusion

The abnormally overexpression of musashi1 probably contributes to HCC development. Besides, elevated expression of musashi1 predicts a poor prognosis in patients with HCC which could be a promising candidate for clinical treatment of HCC.

## Supplementary information

**Additional file 1: Figure S1.** Transfection efficiency of shRNA targeting musashi1. Protein expression levels of musashi1 transfected with various shRNAs targeting musashi1 in SMMC-7721. sh, short hairpin.

## Data Availability

The datasets used and/or analyzed during the current study are available from the corresponding author on reasonable request.
