# Supplementary figures and images for: Upregulation of musashi1 increases malignancy of hepatocellular carcinoma via the Wnt/β-catenin signaling pathway and predicts a poor prognosis
Source: BMC Gastroenterol. 2019 Dec 30;19:230. doi: 10.1186/s12876-019-1150-6 (PMC6937928; doi:10.1186/s12876-019-1150-6)

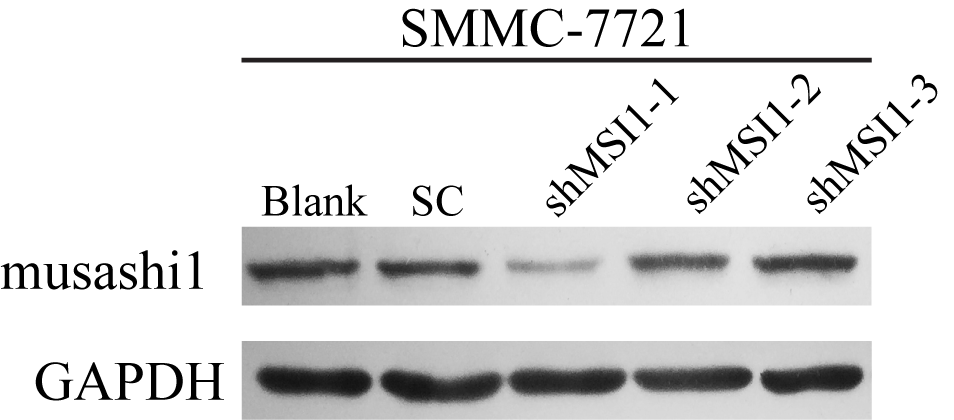

Supplement: Supplementary file 1 — Additional file 1: Figure S1. Transfection efficiency of shRNA targeting musashi1. Protein expression levels of musashi1 transfected with various shRNAs targeting musashi1 in SMMC-7721. sh, short hairpin. [file 12876_2019_1150_MOESM1_ESM.tif]
